# Supplementary material for: Tumor-Specific EphA2 Receptor Tyrosine Kinase Inhibits Anti-Tumor Immunity by Recruiting Suppressive Myeloid Populations in Murine Models of Non-Small Cell Lung Cancer
Source: Cancers (Basel). 2025 Aug 19;17(16):2693. doi: 10.3390/cancers17162693 (PMC12384598; doi:10.3390/cancers17162693)
Supplement: Supplementary file 1 [file cancers-17-02693-s001.zip › cancers-3746537-supplementary figures and tables.pdf]

Supplementary Materials: Tumor-Specific EphA2 Receptor Tyrosine Kinase Inhibits Anti-tumor Immunity by Recruiting Suppressive Myeloid Populations in Murine Models of Non-Small Cell Lung Cancer

Eileen Shiuan, Shan Wang and Dana M. Brantley-Sieders

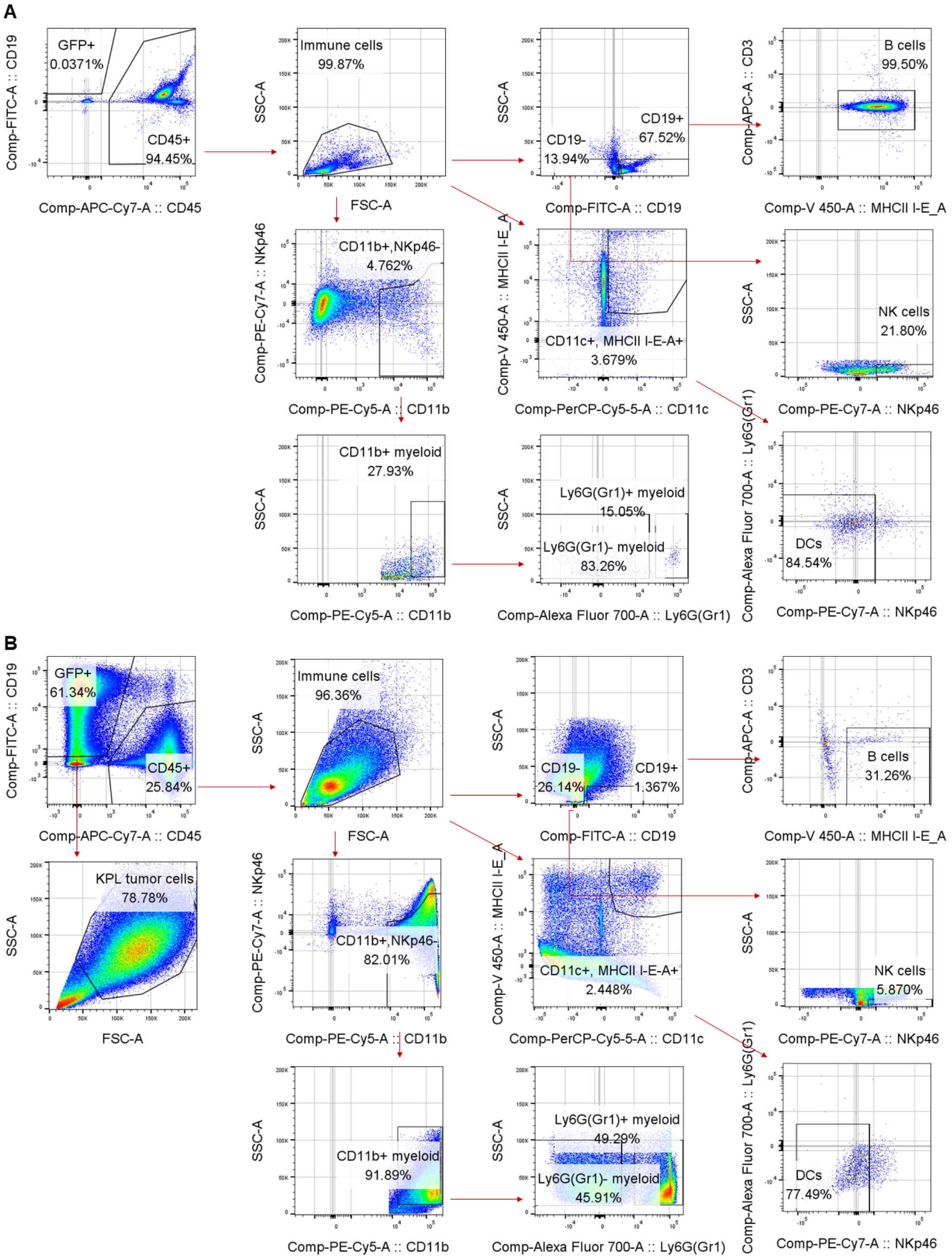

Figure S1. Flow cytometry gating strategy in nude mice. Representative flow plots starting from live single cell population for draining lymph node (A) and tumor (B).

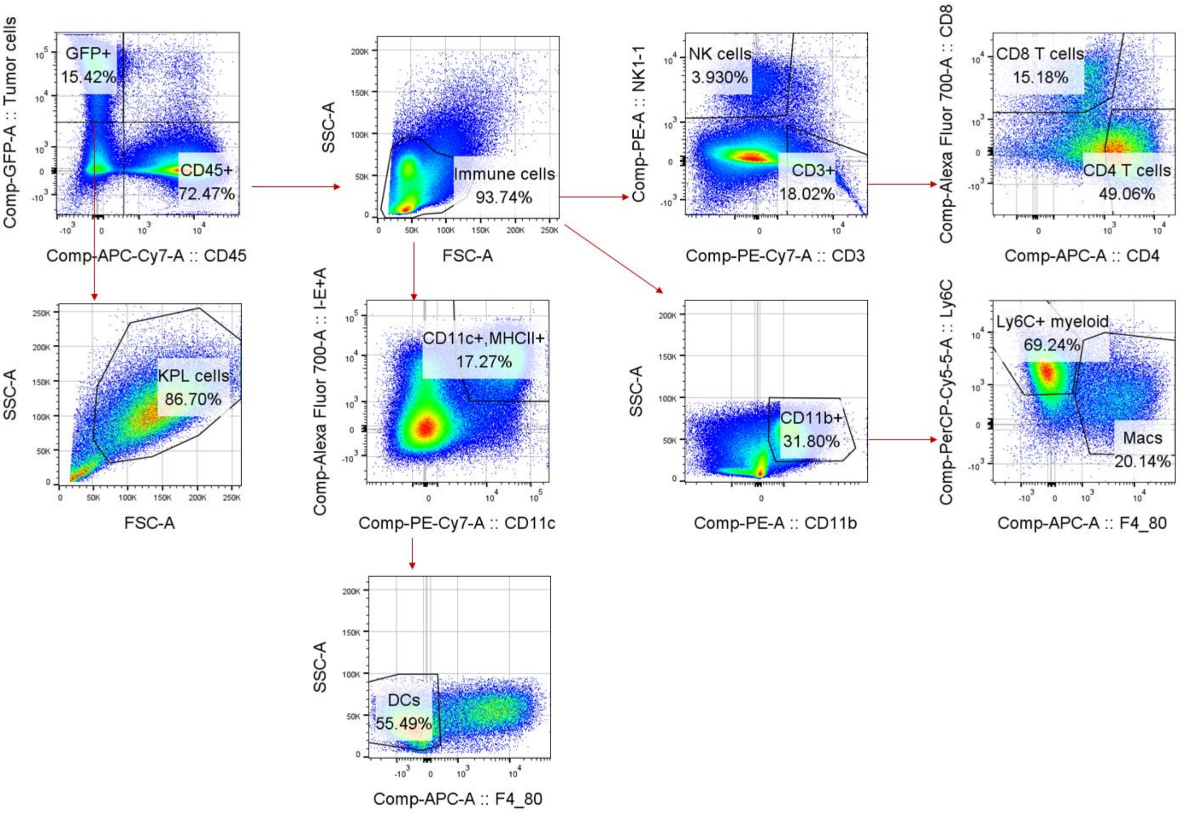

Figure S2. Flow cytometry gating strategy in C57BL/6 mice. Representative flow plots starting from live single cell population for tumor-bearing lungs.

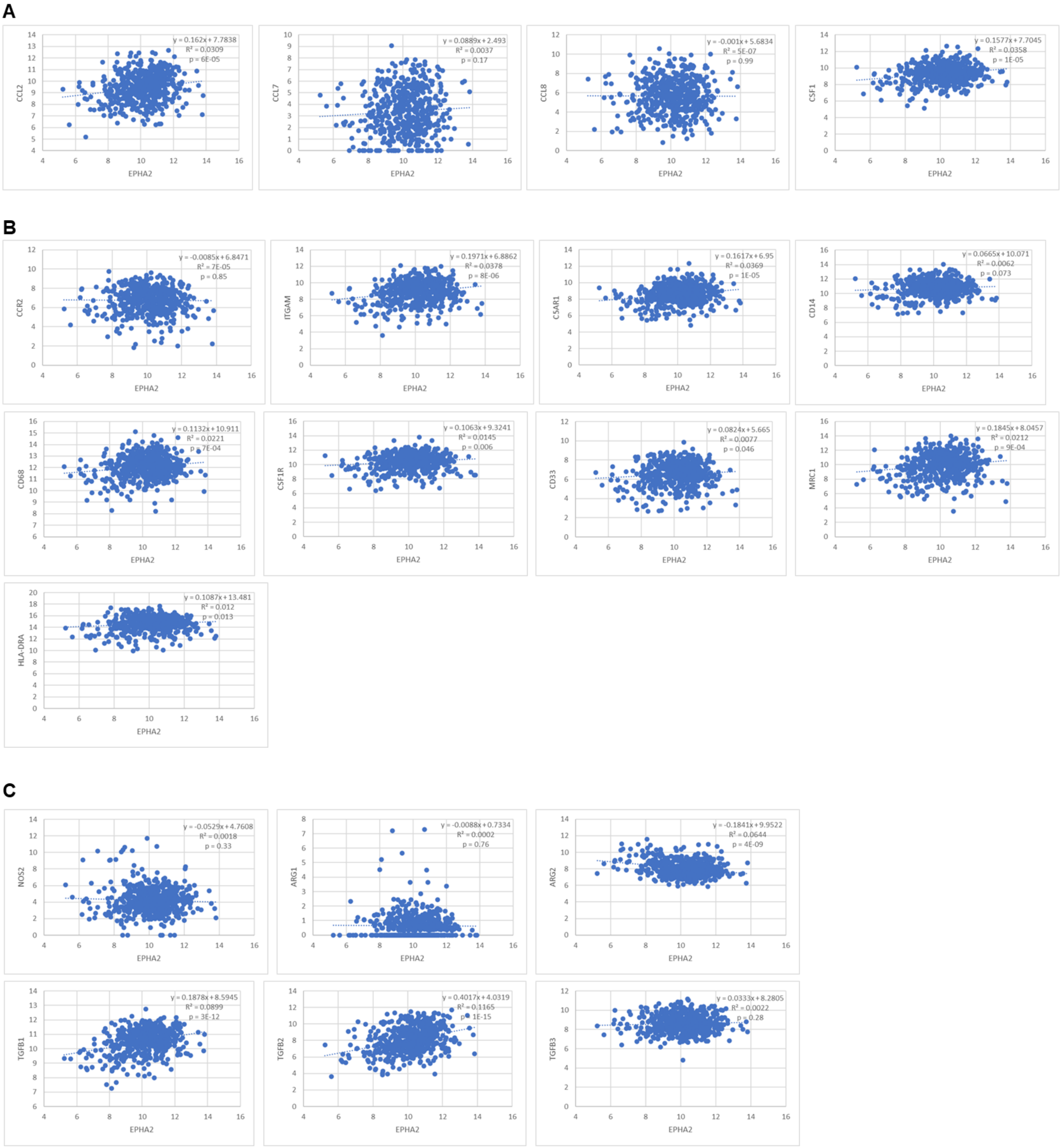

Figure S3. Expression of EPHA2 does not strongly correlate with markers of suppressive myeloid populations in human lung adenocarcinoma. (A) Correlation of transcript abundance (log2(TPM+1)) for *EPHA2* with myeloid chemokines and growth factor, as well as (B) myeloid surface markers and (C) immunosuppressive proteins in primary tumor samples from the TCGA LUAD dataset (n = 517).

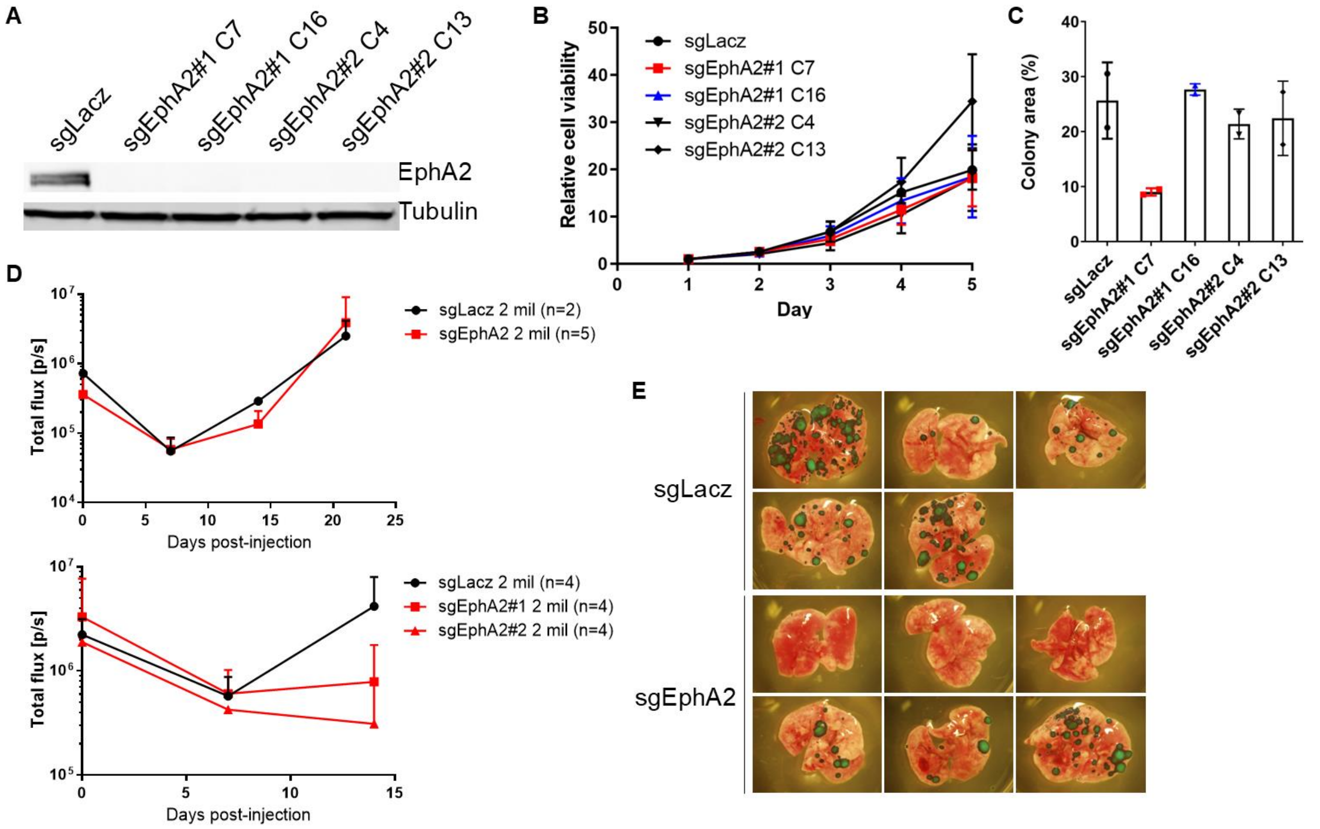

Figure S4. EphA2 knockout does not significantly impact KPL tumor cell proliferation and tumor growth. (A) Confirmation of EphA2 expression knockout via CRISPR/Cas9 in several KPL cell clones by western blot. (B, C) In vitro cell viability of KPL cells with control sgLacz and sgEphA2 by MTT (n = 6) and colony formation assays (n = 2). (D) Quantification of bioluminescence signal at indicated time points of lung tumors formed via tail vein injection of control versus pooled sgEphA2 KPL cells. (E) Representative gross specimens of GFP+ KPL tumor-bearing lungs on day 21 after tail vein injection. Data shown are averages ± SD.

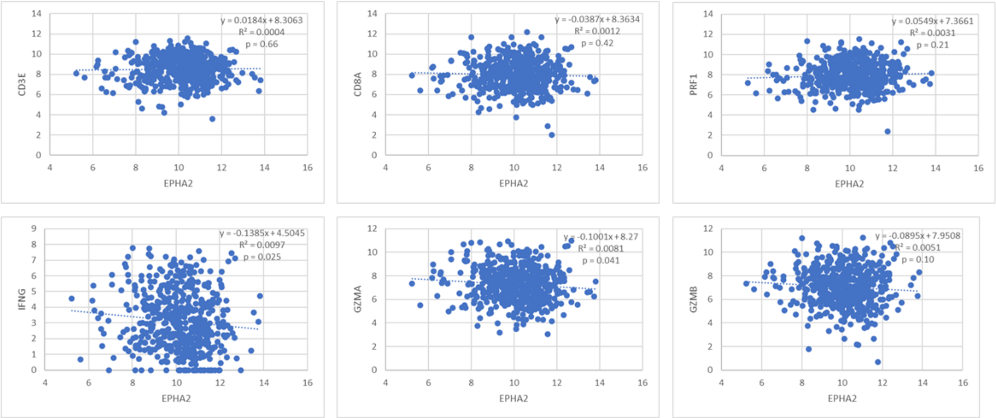

Figure S5. Expression of EPHA2 does not strongly correlate with markers of cytotoxic T-cell populations in human lung adenocarcinoma. Correlation of transcript abundance (log2(TPM+1)) for *EPHA2* with markers of cytotoxic T cells in primary tumor samples from the TCGA LUAD dataset (n = 517).

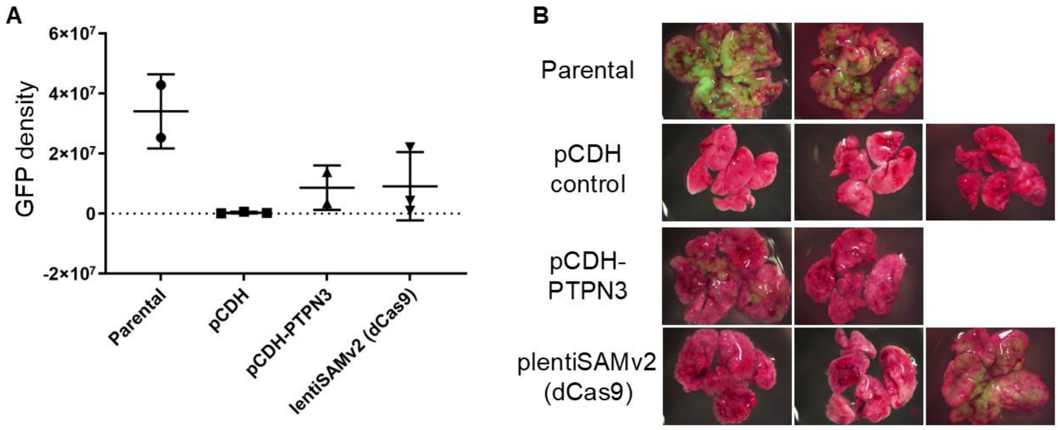

Figure S6. Additional overexpression experiments. (A) Quantification of GFP density of lung tumors 14 days after tail vein injection. (B) Representative gross specimens of GFP+ KPL tumor-bearing lungs. Data shown are averages ± SD.

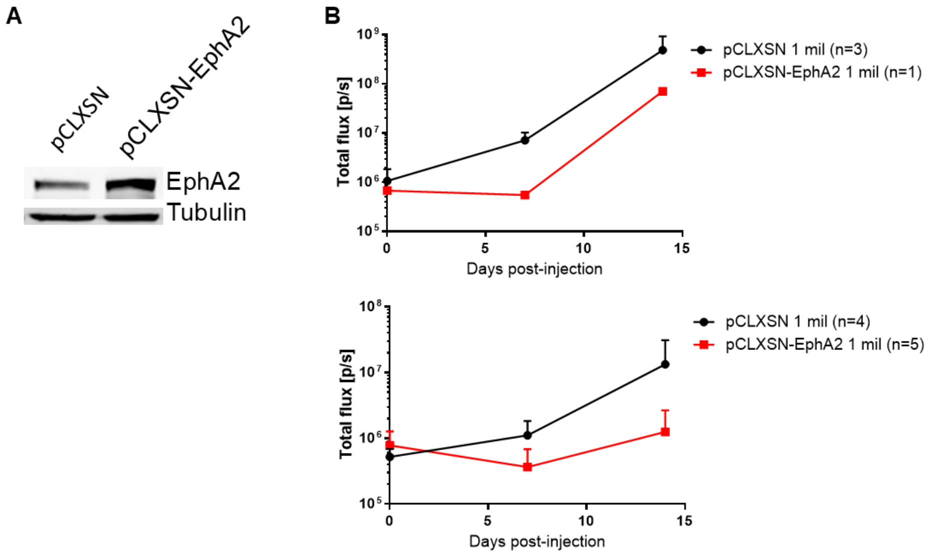

Figure S7. EphA2 overexpression using retroviral transduction did not recapitulate in vivo results using lentiviral transduction. (A) Confirmation of EphA2 overexpression using retroviral transduction in KPL cells by western blot. (B) Quantification of bioluminescence signal at indicated time points of lung tumors formed via tail vein injection of control EphA2-overexpressing KPL cells. Data shown are averages ± SD.

Table S1. Antibodies used in flow cytometry analysis.

Antibody target	Manufacturer	Catalog #	Fluorophore	Dilution	RRID
**MHCII I-E/A**	Tonbo Biosciences	75-5321	V450	1/250	AB_2621965
**CD8a**	BD Biosciences	560469	V450	1/250	AB_1645281
**CD45.2**	Tonbo Biosciences	35-0454	V450	1/500	AB_2621965
**CD19**	Tonbo Biosciences	35-0193	FITC	1/250	AB_2621682
**CD3e**	BD Biosciences	553061	FITC	1/250	AB_394594
**CD44**	Tonbo Biosciences	35-0441	FITC	1/250	AB_2621688
**NK1.1**	BD Biosciences	557391	PE	1/250	AB_396674
**CD11b**	eBiosciences	12-0112-82	PE	1/500	AB_2734869
**CD25**	eBiosciences	12-0251-81	PE	1/500	AB_465606
**CTLA-4**	BD Biosciences	561718	PE	1/250	AB_10895585
**CD3e**	BD Biosciences	561826	APC	1/500	AB_10896663
**CD4**	Biolegend	100516	APC	1/1000	AB_312719
**F4/80**	eBiosciences	17-4801-82	APC	1/250	AB_2784648
**CD3e**	Tonbo Biosciences	65-0031	PerCP-Cy5.5	1/250	AB_2621872
**CD11b**	Tonbo Biosciences	65-0112	PerCP-Cy5.5	1/500	AB_2621885
**CD11c**	BD Biosciences	560584	PerCP-Cy5.5	1/250	AB_1727422
**F4/80**	eBiosciences	45-4801-82	PerCP-Cy5.5	1/250	AB_914345
**Ly6C**	eBiosciences	45-5932-82	PerCP-Cy5.5	1/250	AB_2723343
**Ly6G(Gr1)**	eBiosciences	45-5931-80	PerCP-Cy5.5	1/500	AB_906247
**Ly6G(Gr1)**	Tonbo Biosciences	80-5931	rF710	1/1000	AB_2621999
**CD8a**	Tonbo Biosciences	80-0081	rF710	1/500	AB_2621977
**MHCII I-E/A**	Biolegend	107622	Ax700	1/1000	AB_493727
**PD-1**	BD Biosciences	565815	APC-R700	1/500	AB_2739366
**CD4**	Tonbo Biosciences	55-0041	PE-Cy5	1/2500	AB_2621816
**CD11b**	Tonbo Biosciences	55-0112	PE-Cy5	1/5000	AB_2621818
**CD3e**	BD Biosciences	552774	PE-Cy7	1/750	AB_394460
**NKp46**	eBiosciences	25-3351-82	PE-Cy7	1/750	AB_2573442
**CD11c**	BD Biosciences	561022	PE-Cy7	1/500	AB_2033997
**CD69**	BD Biosciences	552879	PE-Cy7	1/500	AB_394508
**CD25**	Tonbo Biosciences	60-0251	PE-Cy7	1/500	AB_2621843
**CD45**	BD Biosciences	557659	APC-Cy7	1/500	AB_396774

Table S2. Gating strategy used in flow cytometry analysis.

Cell population	Gating strategy
**KPL cells**	CD45-, GFP+, SSA^{hi}
**Immune cells**	CD45+, GFP-, $SSA^{lo/int}$
**NK cells (nude mice)**	SSC^{lo}, CD19-, NKp46+
**B cells (nude mice)**	SSC^{lo}, CD19+, MHCII+, CD3-
**Gr1+ myeloid cells (nude mice)**	SSC^{int}, $CD11b^{hi}$, Ly6G(Gr1)+, MHCII-
**Gr1- myeloid cells (nude mice)**	SSC^{int}, $CD11b^{hi}$, Ly6G(Gr1)-
**Macrophages (nude mice)**	SSC^{int}, $CD11b^{hi}$, F4/80+, Ly6G(Gr1)-
**DCs (nude mice)**	CD11c+, MHCII+, NKp46-, Ly6G(Gr1)-
**CD4+ T cells**	CD3e+, NK1.1-, CD4+, CD8a-
**CD8+ T cells**	CD3e+, NK1.1-, CD4-, CD8a+
**NK cells**	CD3e-, NK1.1+
**Macrophages**	SSC^{int}, $CD11b^{hi}$, F4/80+, Ly6C-
**Monocytes**	SSC^{int}, $CD11b^{hi}$, F4/80-, Ly6C+
**DCs**	CD11c+, MHCII+,F4/80-

Table S3. TaqMan RT-PCR probes.

Target gene	Assay ID
**Actb**	Mm02619580_g1
**Arg1**	Mm00475988_m1
**C5ar1**	Mm00500292_s1
**Ccl2**	Mm00441242_m1
**Ccl7**	Mm00443113_m1
**Ccl8**	Mm01297183_m1
**Ccl12**	Mm01617100_m1
**Ccr2**	Mm99999051_gH
**Csf1r**	Mm01266652_m1
**Tgfb1**	Mm01178820_m1
**Tgfb2**	Mm00436955_m1
**Tgfb3**	Mm00436960_m1
