# Supplementary figures and images for: Tumor-Specific EphA2 Receptor Tyrosine Kinase Inhibits Anti-Tumor Immunity by Recruiting Suppressive Myeloid Populations in Murine Models of Non-Small Cell Lung Cancer
Source: Cancers (Basel). 2025 Aug 19;17(16):2693. doi: 10.3390/cancers17162693 (PMC12384598; doi:10.3390/cancers17162693)

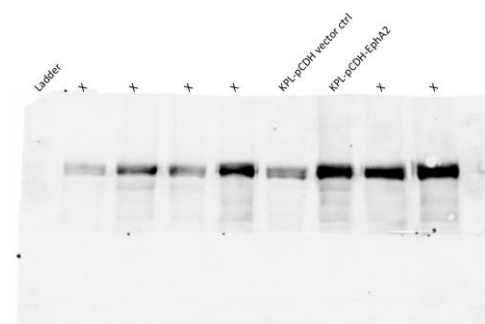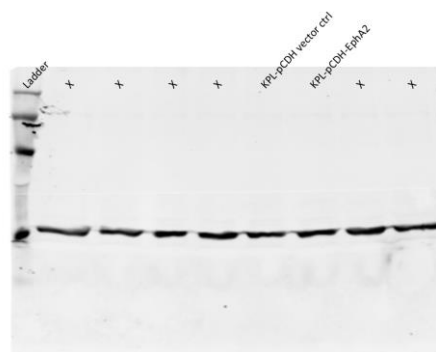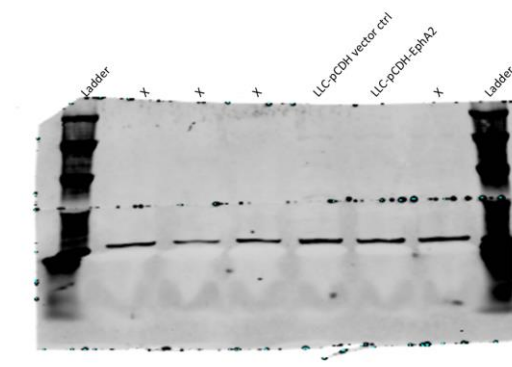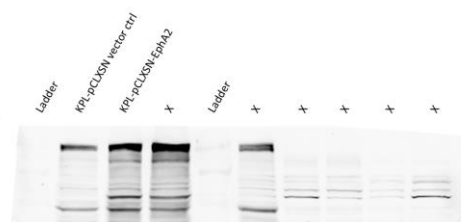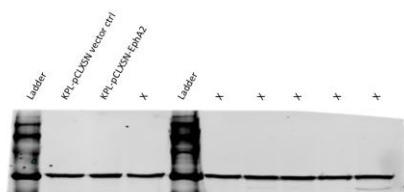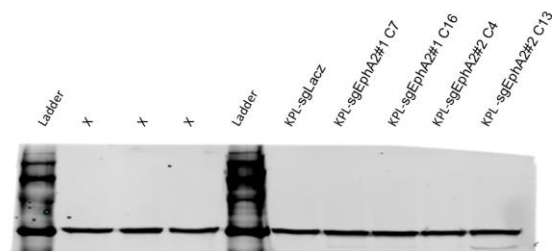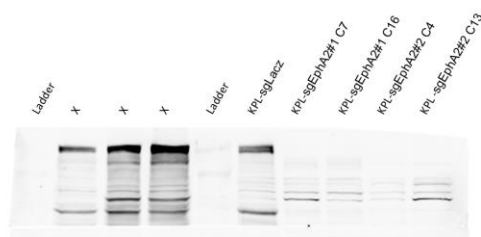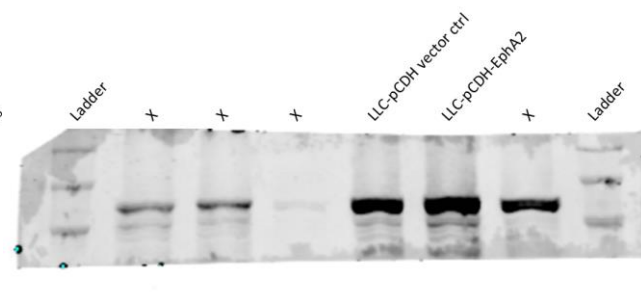

Document S1. Original Western Blots.

Supplement: Supplementary file 1 [file cancers-17-02693-s001.zip › cancers-3746537-Document S1.pdf]
